# Supplementary material for: Interactions of SARS-CoV-2 with Human Target Cells—A Metabolic View
Source: Int J Mol Sci. 2024 Sep 16;25(18):9977. doi: 10.3390/ijms25189977 (PMC11432161; doi:10.3390/ijms25189977)
Supplement: Supplementary file 1 [file ijms-25-09977-s001.zip › ijms-3185089-supplementary.docx]

Supplementary Figure S1


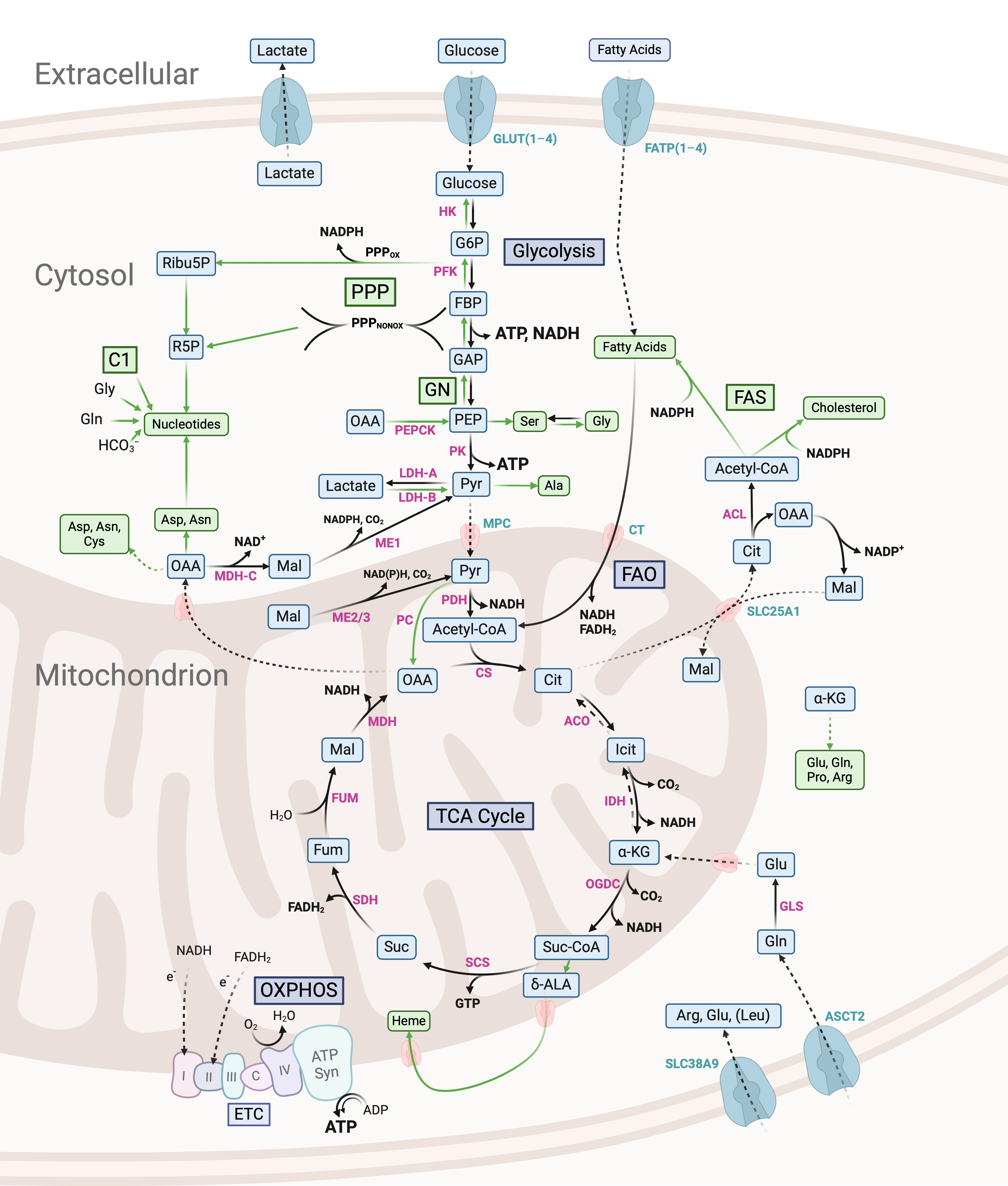


**Figure S1.** (created with BioRender.com): Overview of the major catabolic and anabolic pathways of human cell metabolism. The catabolic pathways mainly consist of glycolysis, the tricarboxylic acid cycle (TCA cycle), fatty acid oxidation (FAO), and degradation of certain amino acids, especially Gln (*via* glutaminolysis). These pathways degrade (by oxidation) essential nutrients, like glucose, fatty acids, and Gln, thereby generating energy and important intermediates that serve as precursors for the anabolic pathways, leading to the biosynthesis of nucleotides, the self-produced amino acids [i.e. Ala, Arg, Asn, Asp, Glu, Gln, Cys, Gly, Pro, Ser, and Tyr (from Phe)], and fatty acids/lipids. Gluconeogenesis (GN) and the pentose-phosphate pathway (PPP) also belong to the anabolic branch. GN is essentially based on the reversal of glycolysis. PPP branches off the glycolytic pathway from glucose-6-phosphate (G6P) and consists of an oxidative (PPP_OX_) and a non-oxidative arm (PPP_NONOX_). The oxidative arm generates the reducing equivalent NADPH which is necessary for fatty acid synthesis (FAS). PPP_NONOX_ delivers intermediates for other biosynthetic pathways, e.g. ribose-5-phosphate (R5P) for the biosynthesis of nucleotides, erythrose-4-phosphate, and sedoheptulose-7-phosphate. Energy in form of ATP is mainly obtained *via* substrate phosphorylation in the glycolytic pathway and *via* oxidative phosphorylation (OXPHOS) in the electron transport chain (ETC). The oxidation of glucose to pyruvate (Pyr) in glycolysis requires NAD (thereby converted to NADH/H+) and yields 2 ATP per glucose molecule. In OXPHOS, electrons from NADH/H^+^ and FADH_2,_ generated in glycolysis and the TCA cycle, respectively, are transferred to oxygen in ETC yielding ATP and H_2_O. This process is a highly efficient energy-delivering process generating up to 36 ATP per glucose molecule, but the rate of ATP production in OXPHOS is quite slow compared to that of glycolysis. A faster ATP production is achieved by “aerobic glycolysis” (also known as “Warburg effect“) [311], which can be caused by the activation of pyruvate kinase isoenzyme 2 (PKM2). The last step of glycolysis is the conversion of phosphoenolpyruvate (PEP) into Pyr by pyruvate kinase (PK), which exists in two isoforms pyruvate kinase isoenzyme 1 (PKM1) and PKM2 [312]. PKM1, the major form functional in normal glycolysis, is enzymatically more active and promotes the entry of Pyr into the mitochondrial TCA cycle. Dimeric PKM2, the major form in aerobic glycolysis, displays low enzymatic activity, thereby slows down the conversion of PEP to Pyr and instead promotes the cytosolic accumulation of metabolic intermediates of the glycolytic pathway and leads to the formation of lactate [313]. The reduction of Pyr to lactate is performed in the cytoplasm by the NADH/H^+^-dependent lactate dehydrogenase A (LDH-A). In this reaction, NAD is regenerated, which can be used for the further oxidation of glucose in glycolysis. In addition, aerobic glycolysis also fuels the PPP. The formed lactate is subsequently excreted from the cells to avoid toxic effects by lactate-mediated cell acidification. But even in cells using aerobic glycolysis for rapid ATP production (e.g. in most activated immune cells [314,315], cancer cells and established cell lines deriving from cancer cells [316,317]), reactions of the mitochondrial TCA cycle, generating intermediates for anabolic pathways cycle are still functioning [318]. This is made possible with the help of anaplerotic reactions, including especially the carboxylation of Pyr to oxaloacetate (OAA) by pyruvate carboxylase (PC) and the conversion of Glu to α-ketoglutarate (α-KG) by glutamate dehydrogenase. The central catabolic pathways (glycolysis, TCA cycle, FAO, OXPHOS) are indicated by dark blue boxes and major intermediates of these pathways by light blue boxes. Anabolic pathways, including biosynthesis of amino acids, nucleotides, fatty acids (FAS) and lipids, as well as PPP and gluconeogenesis (GN) and their end products are indicated by green arrows and boxes. Dotted arrows indicate transporters (named in cyan), regular arrows indicate enzymatic steps (enzymes named in pink).

Supplementary Figure S2


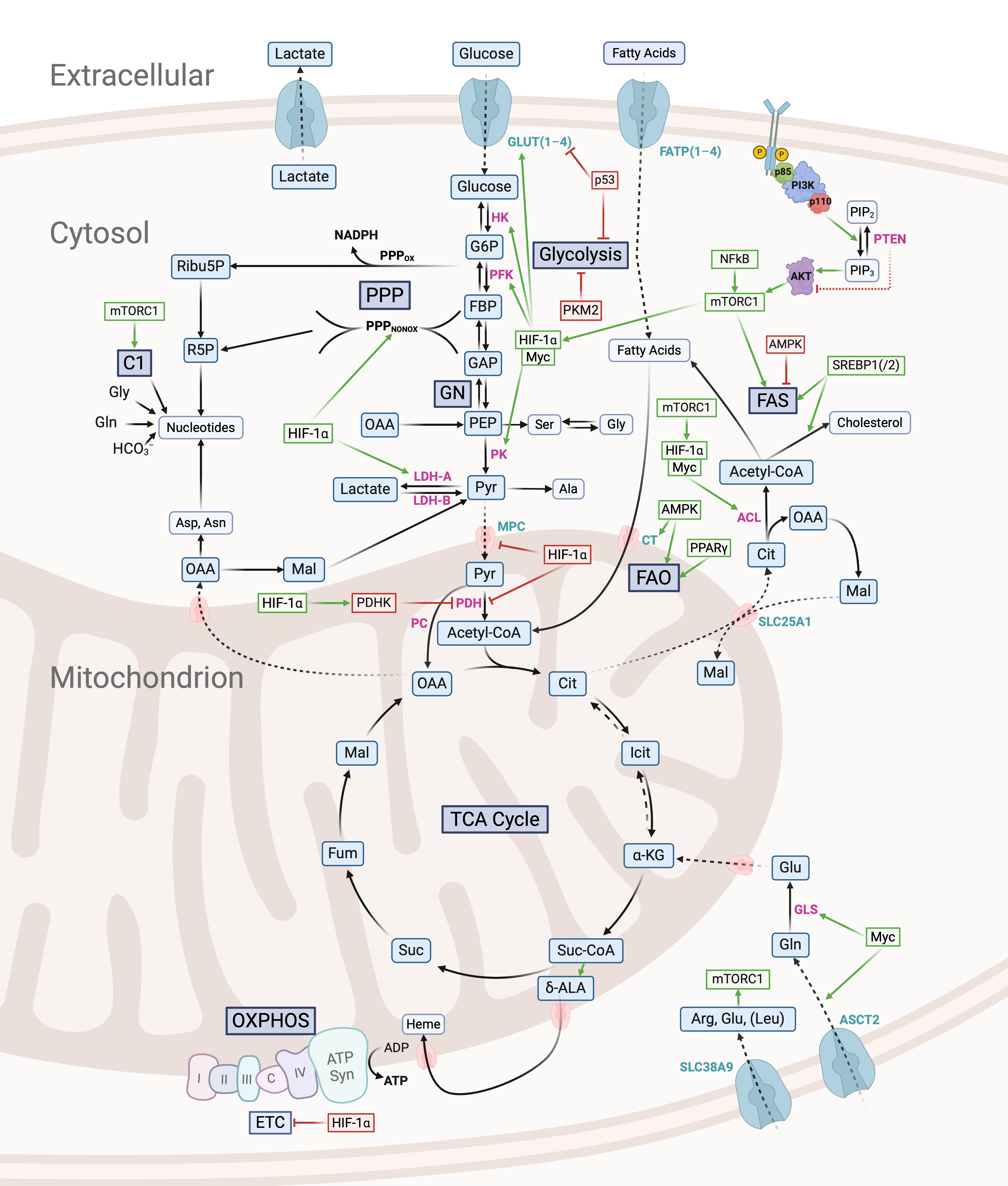


**Figure S2.** (created with BioRender.com): Regulation of the major metabolic pathways by regulatory factors. This regulatory network (only partly shown) consists of (i) external membrane-bound receptors sensing different environmental conditions, such as the external concentration of some nutrients (e.g. glucose and amino acids) and growth hormones, (ii) transcription factors in particular the “nuclear factor kappa-light-chain-enhancer of activated B-cells” (NF-kB), the activator protein 1 (AP-1), the nuclear factor of activated T-cells (NFAT) [319–322], and several oncogenes and tumor suppressors, and (iii) several signaling factors and pathways involved in the regulation of the central metabolism, especially the PI3K/Akt/mTOR and HIF-1 pathways. TORC1 is a central master regulator of cell growth and viability and maintains the cellular balance between anabolism and catabolism in response to environmental factors (e.g. growth factors, amino acids, stress factors). Activated mTORC1 enhances nucleotide synthesis *via* activation of the NADP^+^-dependent methylenetetrahydrofolate dehydrogenase 2 (MTHFD2), lipogenesis and cholesterol biosynthesis *via* activation of the sterol regulatory element-binding proteins SREBP1 and SREBP2, key transcription factors that regulates the expression of genes involved in lipogenesis and cholesterol synthesis [323,324]. Moreover, activated mTORC1 enhances the uptake of glucose and glycolysis *via* activation of the hypoxia-inducible transcription factor 1 (HIF-1) [325,326]. Indeed, HIF-1 is important for the cellular adaptation to hypoxia and to oxygen radicals [95,327]. Regulation of the metabolism by activated HIF-1 is a complex process that includes the up- or down-regulation of numerous genes involved in different metabolic pathways, in particular several genes whose products participate in glucose uptake and glycolysis, including the GLUT1 transporter and enzymes such as LDH-A and pyruvate dehydrogenase kinase 1 (PDHK1). The latter enzyme promotes glycolysis, but inhibits formation of acetyl-CoA and hence its entry into the TCA cycle, and thus mitochondrial respiration [328–331]. Several oncogenes (e.g. the myelocytomatosis oncogene Myc and Ras proteins) and tumor suppressors [e.g. p53, the phosphatase and tensin homolog (PTEN), and the liver kinase B1 (LKB1/AMPK)] function as transcriptional regulators of metabolism [332–336]. They act at various metabolic key nodes [337–342], and normally prevent excessive catabolic and/or anabolic metabolism. Certain missense and knock-out mutations in the genes encoding these factors (frequently observed in p53 and Myc) lead to the activation of various catabolic pathways [56,343]. Myc regulates the expression of genes for the glucose transporters GLUT1 and GLUT4, and for key glycolytic enzymes, such as hexokinase (HK), phosphofructokinase (PFK), and pyruvate kinase (PK). It also promotes Gln uptake and glutaminolysis, and thereby replenishes the TCA cycle [344,345]. Mutations or certain physiological conditions, leading to enhanced Myc concentration or activity, turn Myc into an oncoprotein (observed in many cancers) which causes enhanced glucose uptake, glycolysis and glutaminolysis, a process known as “Warburg effect“ [346–348]. AMPK, induced by an increased AMP/ATP ratio, triggers catabolic and inhibits anabolic processes, e.g., it stimulates (upon glucose shortage) the uptake of fatty acids into mitochondria and their subsequent FAO, whereas acetyl-CoA carboxylase activity is inhibited by activated AMPK, thus blocking FAS [349]. Activated AMPK also reduces global protein synthesis by antagonizing the kinase activity of mTORC1. For details, see also text and legend of Figure S1. The major activating regulators of the different metabolic steps are marked by green-boxes and green arrows, while regulators inhibiting pathways are marked by red boxes and red signs.
